# *Lactobacillus* spp. create a protective micro-ecological environment through regulating the core fucosylation of vaginal epithelial cells against cervical cancer

**DOI:** 10.1038/s41419-021-04388-y

**Published:** 2021-11-20

**Authors:** Qingjie Fan, Yuanhang Wu, Mechou Li, Fan An, Lulu Yao, Meixian Wang, Xiuying Wang, Jieli Yuan, Kui Jiang, Wenzhe Li, Ming Li

**Affiliations:** 1 College of Basic Medical Science, Dalian Medical University, Dalian, China; 2 Department of Oncology, The First Affiliated Hospital of Dalian Medical University, Dalian, China; 3 Department of Medical Oncology, The Second Affiliated Hospital of Dalian Medical University, Dalian, China; 4 The Cancer Stem Cell Research Institute of Dalian Medical University, Dalian, China; 5 The Reproductive and Genetics Center of Dalian Women and Children’s Medical Center (Group), Dalian, China; 6 The Gynecology and Oncology Ward of Dalian Maternal and Child Health Hospital, Dalian, China

**Keywords:** Cervical cancer, Gynaecological cancer

## Abstract

Vaginal dysbiosis often occurs in patients with cervical cancer. The fucosylation of mucosal epithelial cells is closely related to microbial colonization, and play an important role in protecting the vaginal mucosal epithelial cells. However, no reports on the relationship between vaginal dysbiosis and abnormal mucosal epithelial cell fucosylation, and their roles in the occurrence and development of cervical cancer are unavailable. Here we report that core fucosylation levels were significantly lower in the serum, exfoliated cervical cells and tumor tissue of cervical cancer patients. Core fucosyltransferase gene (Fut8) knockout promoted the proliferation and migration of cervical cancer cells. In patients with cervical cancer, the vaginal dysbiosis, and the abundance of *Lactobacillus*, especially *L. iners*, was significantly reduced. Meanwhile, the abundance of *L.iners* was positively correlated with core fucosylation levels. The *L. iners* metabolite lactate can activate the Wnt pathway through the lactate-Gpr81 complex, which increases the level of core fucosylation in epidermal cells, inhibiting the proliferation and migration of cervical cancer cells, and have application prospects in regulating the vaginal microecology and preventing cervical cancer.

## Introduction

Cervical cancer is the fourth most common malignant tumor in females, with that ~570,000 new cases and 311,000 deaths occur worldwide each year [1, 2]. In developing countries, cervical cancer also presents a high incidence and mortality [3, 4]. Therefore, clarifying the mechanisms by which cervical cancer occurs and develops and seeking effective intervention measures have become urgent issues that remain to be solved.

In recent years, research on the pathogenesis of cervical cancer has become more in-depth. Human papilloma virus (HPV), especially high-risk HPV strains such as HPV16 and 18, is a pathogenic factor recognized to be involved in the onset of cervical cancer [5–7]. However, HPV is only one factor in the pathogenesis of cervical cancer. The combination of multiple factors, such as disorder of the vaginal microbiota in the vaginal microenvironment, an increase in immune inflammatory factors, and an increase in pH, promote the transformation of normal cells to cancerous cells [8]. Vaginal micro-ecological homeostasis is of great importance for maintaining female health. *Lactobacillus* spp. are the most common bacteria in female vagina. They use carbohydrates on host mucosal epithelial cells as an energy source to produce lactic acid to inhibit the adhesion, colonization and growth of pathogenic bacteria [9–11]. Current studies have found that *Lactobacillus crispatus*, *Lactobacillus gasseri*, *Lactobacillus iners*, and *Lactobacillus jensenii* are the most common *Lactobacillus* species in the vaginas of healthy females [12, 13], while patients with cervical cancer often exhibit disorder of the vaginal microbiota, manifested as a significant decrease in the abundance of *Lactobacillus* spp. and significant increase in the abundance of pathogenic bacteria such as *Gardnerella* spp. and *Sneathia* spp [14–16]. Some bacteria such as *Sneathia* spp. and *Fusobacterium* spp. exist only in the vaginas of cervical cancer patients and can be used as diagnostic markers for cervical cancer [17], indicating that disorder of the vaginal microbiota plays an important role in the occurrence and development of cervical cancer. However, the role of vaginal dysbiosis in the pathogenesis of cervical cancer needs to be further studied. Dysbiosis of the vaginal microbiota can induce DNA damage in the local microenvironment, an immune inflammatory response, viral infection, and immune escape. Cooperation between these factors promotes the transformation of normal cells to cancerous cells [18].

The fucosylation of mucosal epithelial cells is closely related to the colonization of microorganisms [19]. α-1,6 Fucosyltransferase (Fut8), the most important fucosyltransferase in mammalian cells, can catalyze the transfer of GDP-β-L-fucose to N-acetylglucosamine (GlcNAc) moieties adjacent to asparagine (Asn) residues in the sugar chain of core fucose [20, 21], which is involved in the occurrence and development of liver cancer, lung cancer, colon cancer and other tumors [22, 23]. Notably, some studies have found that fucosylation tends to be decreased in patients with cervical cancer compared to healthy volunteers [24, 25]. However, to date, the correlation and relationship between abnormal mucosal epithelial cell fucosylation and disorder of the vaginal microbiota and their roles in the occurrence and development of cervical cancer have not been reported.

In this study, we collected vaginal swabs, serum, and exfoliated cervical cells from healthy volunteers and cervical cancer patients to systematically analyze and compare their vaginal microbiota and core fucosylation levels. Correlation analysis between the differential microbiota and proteofucosylation levels was performed using gene knockout in cell lines and animal models to explore the related molecular mechanisms. This research provides new insight into the pathogenesis, prevention, and treatment of cervical cancer.

## Materials and methods

### Subjects and sample collection

The study was approved by the ethical committees of Dalian Medical University, Dalian, China. The enrolled healthy controls and patients have filled out the informed consent (V01,2018.4.1) form before sample collection.

A total of 54 healthy women and 65 cervical cancer patients were recruited from the Second Hospital of Dalian Medical University, Dalian, China (Table 1). The diagnosis of cervical squamous carcinoma is based on clinical manifestations, serology, TCT and histopathology. Patients with autoimmune diseases, weakened immune functions, endocrine diseases or diabetes, women taking corticosteroids, antibiotics, imidazole or probiotics, women who have used vaginal preparations in the last month, and patients receiving chemotherapy and radiotherapy were excluded from the study. For all study participants, record the medical history, collect samples of vaginal secretions using sterile cotton swabs during the vaginal examination, and test the vaginal pH. The vaginal swab samples were collected from each woman and immediately stored at −20 °C until transfer to the laboratory on dry ice and then stored at −80 °C before use. The serum samples of 20 healthy volunteers and 35 patients with cervical squamous cell carcinoma were collected. Five exfoliated cell samples and 8 cervical tissue biopsy samples were collected from 5 healthy volunteers, 5 patients with cervical squamous cell carcinoma and 3 patients with cervical squamous cell carcinoma, respectively. The Ethics Hospital Committee of the Second Affiliated Hospital of Dalian Medical University approved this study (No. 2019031).

**Table 1:** Characteristics of the study population.

Variables	Healthy controls	Patients with CESC	*p*-value
Sample size	*n* = 54	*n* = 65	
Age	46.81 ± 8.15	48.65 ± 6.873	0.2950
pH (*n* (%))			<0.001
≤4.5	51 (94.44)	5 (7.69)	
>4.5	3 (5.45)	60 (92.31)	
BMI (kg/m^2^)	24.57 ± 3.427	24.95 ± 4.225	0.5631
HPV status			
HPV negative	7 (12.73)	2 (3.08)	0.0460
HPV16 positive	46 (83.64)	60 (92.31)	0.1428
HPV18 positive	1 (1.82)	6 (9.23)	0.0856
Other high-risk^a^	24 (43.64)	20 (30.77)	0.1475
Low-risk	6 (10.91)	7 (10.77)	0.8139
HPV risk profile			
Single high-risk	25 (45.45)	38 (58.46)	0.1577
Multiple high-risk	23 (41.82)	25 (38.46)	0.7113
High and low-risk	5 (9.09)	7 (10.77)	0.7625
Histology (*n*)			
Squamous cell carcinoma		65	
Adenocarcinoma		-	
Stage (*n*)			
I		31 (47.69)	
II		27 (41.54)	
III		5 (7.69)	
IV		2 (3.08)	

^a^Other high risk: HPV31,33,52,58,35,39,45,51,56,59,68.

### HPV genotyping assay

Using the extracted vaginal metagenomic DNA as a template, the HPV typing of each clinical sample was determined according to the operating instructions of the HPV typing identification kit (bioPerfectus, China).

### Protein extraction, western blot, and lectin blot analysis

The cervical exfoliated cells suspension was firstly centrifuged at 5000 rpm for 5 min. Add an appropriate amount of protein lysis solution to the cell pellet and lyse it on ice for 15–30 min. Gently shake the mixture during the lysis process to make it fully lysed. After lysis, centrifuge at 12,000 rpm for 10 min, collect the supernatant, use the BCA protein concentration determination kit (Takara Bio, Otsu, Japan) to determine the protein concentration of each sample. For serum samples, directly dilute 50 times by PBS and use BCA method to detect the protein concentration.

The total proteins in serum and cell samples were separated by Sodium dodecyl sulfate-polyacrylamide gel electrophoresis (SDS-PAGE), and were then transfered to the nitrocellulose filter (NC) membrane (Merck Millipore, USA). The membrane was sealed for 1 h at RT using 5% BSA and was subsequently incubated overnight at 4 °C with different primary antibodies or Lens Culinaris Agglutinin (LCA, Vector Laboratories). After that, the membrane was incubated with the HRP-conjugated streptavidin or HRP-conjugated Abs (Beyotime) at room temperature (RT) for 1 h, and the protein bands were then visualized by using the ECL kit (Beyotime).

### Mass spectrometry

The serum proteins of patients were separated by SDS-PAGE electrophoresis, and the gel strips were cut into small gel blocks with a size of 1 mm*1 mm, put in EP tubes for washing, decoloring, enzymatic hydrolysis with protease in the gel, and reacting for 15 h in an enzymatic hydrolysis instrument at 37 °C. After the reaction, 400 μL peptide extract (50% CAN, 0.1% TFA) was added immediately, and reacted at 37 °C for 30 min. The product was then transferred to a new EP tube and repeat the peptide extraction process twice. After the reaction was over, it was placed in a freeze dryer and lyophilized. The lyophilized sample was dissolved in 15 μL of 0.1% formaldehyde solution, centrifuged at 20,000 × *g* for 30 min, and 10 μL of the supernatant was taken into a sample bottle. Mass spectrometry analysis is carried out on QExactive or QE HF (thermo), and the chromatographic column is C18, 3 μm, 250 mm × 75 μm (Eksigent). Experimental conditions: 10-h chromatographic gradient, 0.3 μL/min chromatographic flow rate, and the 10 most intense ions in the MS spectrum for analysis. The results obtained were searched in the database using Proteome Discoverer software.

### Immunofluorescence

The cervical cancer tissue samples were fixed overnight and then embedded in paraffin. The paraffin sections were sequentially de-paraffinized and hydrated in xylene, ethanol with different gradient concentrations, and PBS. Immerse the slices in sodium citrate antigen retrieval solution (Beyotime), and react for 30 min in a 95 °C water bath for antigen retrieval. Then, take the slices in 3% H_2_O_2_ solution and incubate at room temperature for 10 min to eliminate endogenous peroxidase. Use 5% BSA to seal the specimens for 1 h at RT. Subsequently, incubate the specimens overnight at 4 °C with β-catenin or LCA. The membrane is incubated with the Alexa Fluor 549 conjugated ChromPure Goat IgG (Jackson) or Andy fluor 488 streptavidin for 1 h at 37 °C, and incubated with DAPI (Solarbio, China) for 10 min at RT. Finally, the slides were observed with a fluorescence microscope (Leica, Germany). The specimens were analyzed by hematoxylin-eosin (H&E) staining. For cell immunofluorescence, use 4% paraformaldehyde to fix and block directly, and the following steps are the same as tissue immunofluorescence.

### Vaginal microbial DNA extraction, PCR amplification and 16S rRNA sequencing

The microbial genomic DNA from the vaginal swab samples samples was extracted using the QlAamp DNA mini Kit (Qiagen). The DNA concentration and purity was measured using the Qubit 2.0 Fluorometer (Thermo Fisher Scientific, USA). PCR was performed to amplify the V3 and V4 region of the bacterial 16 S rRNA gene using the primers 341F (5′-CCTAYGGGRBGCASCAG-3′) and 806R (5′-GGACTACNNGGGTATCTAAT-3′); template DNA was absent in the negative control. PCR products were monitored on a 2% agarose gel. The PCR fragments were sequenced on an Illumina HiSeq platform (Personal Biotechnology Co., Ltd. Shanghai, China). The QIIME software 1.9 package was used to analyze sequences (Quantitative Insights Into Microbial Ecology, http://bio.cug.edu.cn/qiime/). Sequences having a 97% resemblance or higher were categorized as the same operational taxonomic units (OTUs). The alpha diversity of vaginal microbiota was evaluated by the Chao 1 index, the observed species index, and the abundance-based coverage estimator (ACE) index. The beta diversity was evaluated by Principal Component Analysis) (PCA). Linear Discriminant Analysis Effect Size (LEfSe) was used to identify the bacterial taxa differentially represented between groups at different taxonomic levels. A linear discriminant analysis (LDA) was used to estimate the effect size of each deferentially abundant feature (LDA **≥** 4 was shown in figures). The datasets are publicly available (Accession number PRJNA725946).

### Cells and culture conditions

The SiHa cell line (ATCC Number: HTB-35) was obtained from Shanghai Zhong Qiao Xin Zhou Biotechnology Co.,Ltd. China. The HeLa cell line (ATCC Number: CCL-2) was provided by the Cancer Stem Cell Research Institute of Dalian Medical University, China. The 293T cells was obtained from American Type Culture Collection (ATCC ACS-4500, USA). Cell lines were authenticated by STR profiling. SiHa, HeLa, and 293T cells were maintained in Dulbecco’s modification of Eagle’s medium (DMEM, Gibico) with 10% FBS (BI, Gibico), penicillin (60 μg/mL), and streptomycin (100 μg/mL) (Sangon, China) in 5% CO_2_ at 37 °C.

### Establishment of *fut8* gene knockdown and reintroduced cell lines

The pLKO.1 shRNA lentivirus system was used to generate shRNA virus against human Fut8 gene (shFut8). The shFut8 (sense: TCTCAGAATTGGCGCTATG, antisense: CATAGCGCCAATTCTGA- GA, vector: psi-LVRU6GP) and negative control (shctl) were purchased from Genecopoeia (Guangzhou, China). Positive SiHa cells were obtained by puromycine (2 μg/mL) selection.

To prepare Fut8 reintroduced cells, pLHCXsi-U6-Fut8 mutant expression vectors resistant to the siRNAs expressed in shFut8 cells were prepared [26]. The pLHCXsi-U6-Fut8 vector was transduced to Fut8^−/−^ cells by lipofectamine, and positive SiHa cells (Fut8^RE^) were generated after selection with 400 μg/mL hygromycin.

### Cell proliferation assay

Five thousand cells were cultured in a 96-well plate overnight with 5% CO_2_ at 37 °C. Fifty microliters MTT (NJJCBIO, China) was added to each well and incubate at 37 °C for 4 h to reduce MTT to formazan. After incubation, discard the culture medium, add 50 μL dimethyl sulfoxide (DMSO) to each well to fully dissolve the crystals, shake it with a plate shaker, and finally measure the absorbance at 595 nm with a microplate reader (Thermo, Finland).

### Cell scratch test

In all, 5 × 10^5^ SiHa or HiLa cells were inoculated in a 6-well plate to ensure that they can be covered overnight. Scratch vertically and horizontally with the tip of the pipe on the second day, then washed the cells three times with PBS, and added serum-free DMEM medium.

### In vivo tumor growth assay

In all, 1 × 10^7^ of Fut8^+/+^ and Fut8^−/−^ SiHa cells were suspended in 150 μL PBS buffer and injected into six-week-old female athymic nude mice (nu/nu) subcutaneously. The tumor size were measured every week. The nude mice were sacrificed on the 21st day. Tumor volumes were calculated according to the following formula: tumor volume **=** (length) × (width)^2^ × 0.5, and tumor weights were recorded. All the animal works were approved by the Ethics Committee of Dalian Medical University (No. AEE17013).

### Bacterial strains and growth condition

The strain *Lactobacillus iners* ATCC 55195 (*L. iners*) and *Bacteroides fragilis* ATCC 25285 (*B. fragilis*) used in this study were obtained from American Type Culture Collection (ATCC). *L. iners* and *B. fragilis* were grown at 37 °C, 5% CO_2_ on Brain-Heart Infusion (BHI) medium under anaerobic conditions. The growth of bacteria was evaluated by detecting the optical density (OD) at 612 nm.

### Stimulation of SiHa cells by *L. iners* in vitro

10^6^ of SiHa cells were seeded in a 6-well plate, cultured at 37 °C for 24 h, and then divided into control group, treatment group and inhibition group. The control group was added with PBS, and the experimental group was added with the supernatant of *L. iners* culture broth with MOI **=** 1000, stimulated for 30, 60, and 120 min respectively. In the inhibition group, the supernatant of *L. iners* with MOI **=** 1000 was added. After 60 min of stimulation, 10 µL of DKK-1 (Wnt pathway inhibitor) at concentrations of 10^3^, 10^4^, 2 × 10^4^ was added, then use the lection blot and WB methods to determine the core fucosylation and β-catenin expression levels of the SiHa cells, respectively.

### Chromatin immunoprecipitation- real-time fluorescence quantitative PCR (CHIP-qPCR)

Chromatin immunoprecipitation was performed by using the CHIP Assay Kit (Beyotime). In all, 1 × 10^7^ of SiHa cells were inoculated into 10 cm culture dishes, cultured at 37 °C for 24 h, and then the medium was changed. The cells were divided into the control group and the *L*. *iners* group. The L. iners metabolites were incubated with SiHa cells for 30 min at MOI = 1:1000, washed twice with PBS, changed the medium, and incubated at 37 °C for 30 min. The control group was added with the same volume of culture medium and incubated at 37 °C for 60 min. Add 1% formaldehyde to the medium for cross-linking of protein and DNA fragments. Subsequently, the genomic DNA will be broken by ultrasonic disruption. Add anti-β-catenin to the sample to precipitate TCF/β-catenin complex rotate overnight at 4 °C, add 60 µL Protein A + G Agarose/ Sal-mon Sperm DNA the next day. Finally, the obtained precipitate was analyzed by fluorescence real-time quantitative PCR (qPCR) technology following the SYBR Green Pro TaqHS Qpcr Kit instructions (Accurate Biotechnology (Hunan) Co.,Ltd.). The primer sequences used to detect the promoter region of human fut8 gene were as follows: Fut8 promoter F: 5′-CACCCCTTCTTGCTCTTGGC-3′, Fut8 promoter R: 5′-GACTGTCAGCCATGGAAGCAT-3′.

### Dual-luciferase reporter gene assay

The SiHa cells were grown in a 96-well plate at the density of 2.0 × 10^4^ cells per well. Then, Lipofectamine TM 2000 (Invitrogen) was used to co-transfect SiHa cells with 0.2 ng of pGL3- FUT8-WT or pGL3- FUT8-MUT (Wuhan GeneCreate Biological Engineering Co., Ltd.) and 2 ng of pRL-TK (Promega, Madison, Wisconsin) with a TCF-pcDNA3.1. After transfection for 48 h, the relative activity of luciferase was calculated by normalizing firefly luciferase to renilla luciferase. Each experiment was duplicated three times.

### RNA-seq experiments and analysis

Total RNA was extracted from Fut8^+/+^ and Fut8^−/−^ SiHa cells using the RNA Nano 6000 Assay Kit of the Agilent Bioanalyzer 2100 system to check its integrity (Agilent Technologies, CA, USA). Sequencing libraries were generated using NEBNext® UltraTM RNA Library Prep Kit for Illumina® (NEB, USA) following manufacturer’s recommendations. The clustering of different groups of samples was performed on a cBot Cluster Generation System using HiSeq X-Ten/NovaseqS4 PE Cluster Kit (Illumina) according to the manufacturer’s instructions. After cluster generation, the library preparations were sequenced on an Illumina HiSeq X-Ten/NovaseqS4 platform and 150 bp paired-end reads were generated.

For the RNA-seq experiments of SiHa cells upon treatment of *L. iners* metabolites, the SiHa cells were divided into the control group and the *L. iners* group. In the *L. iner* group, the *L. iners* metabolites was added into SiHa cells for 30 min at MOI **=** 1:1000, washed twice with PBS, changed the medium, and incubated at 37 °C for 30 min. The control group was added with the same volume of culture medium and incubated at 37 °C for 60 min.

### Detection of lactate

The vaginal swab samples of healthy volunteers and patients were dissolved in saline, the concentration of lactate in the solution was then detected by using the Lactic Acid assay kit (NJJCBIO, China) following the manufacturer’s guide. The kit was also used to determine the lactic acid content in the metabolites of *L.iners* in the logarithmic growth phase.

### Stimulation of SiHa cell by lactate in vitro

A total of 10^6^ of SiHa cells were seeded in a 6-well plate, cultured at 37 °C for 24 h, and then the medium was changed. According to the completely random method, the cells were divided into four groups: the Control group, *L. iners group*, Lactate (Lac) group and the Lac+3-hydroxybutyrate (3-OBA) group. 3-OBA is an antagonist of lactic acid [27]. They were stimulated for 60 min, and then the proteins were extracted to detect the level of Gpr81, Wnt3, β-catenina (Abcam) and LCA. The fluorescence-labled antibody or LCA were used to determine the levels of β-catenin expression and protein core fucosylation of cells in the four groups, respectively.

### Statistical analysis

All the experiments were presented as mean ± standard error of the mean (SEM, *n* **≥** 3). Except of 16 S rRNA sequence and RNA-seq experiments analysis, the GraphPad Prism Program (Version 9.0; GraphPad Software Inc., La Jolla, CA, USA) was used for statistical analysis and difference comparison of experimental data. A non-parametric *t*-test was performed between the two groups. One-way analysis of variance and Tukey’s test were used between multiple groups to evaluate whether there were statistical differences between the groups. A *p*-value of less than 0.05 was considered statistically significant. *p < 0.05, **p < 0.01, ***p < 0.001, ****p < 0.0001.

## Results

### Cervical cancer patient and healthy female cohorts

In this study, 54 healthy females and 65 female patients with cervical cancer were recruited (Table 1). There was no significant difference in age and BMI between groups (*p* **>** 0.05). Testing of the vaginal pH showed that 94.44% of the healthy females had a vaginal pH ≤ 4.5, while 92.31% of the patients with cervical cancer had a vaginal pH > 4.5 (*p* < 0.001). The samples collected from the cervical cancer patients were mainly stage I and II cervical squamous cell carcinoma, accounting for 89.23% of the patients. Multiple studies have reported that HPV infection is closely related to the occurrence and development of cervical cancer [5–7]. We thus conducted HPV testing of the healthy females and cervical cancer patients and found that 87.27% of the healthy females were infected with HPV, and HPV16, 18 and other high-risk HPV strains accounted for 87.23% of these infections. The HPV infection rate of the cervical cancer patients was 96.92%, and infection with high-risk strains accounted for 88.89% of these infections, but the difference was not significant (*p* **>** 0.05).

### Core fucosylation was significantly reduced in patients with cervical cancer compared to the healthy females

**Fig. 1:**
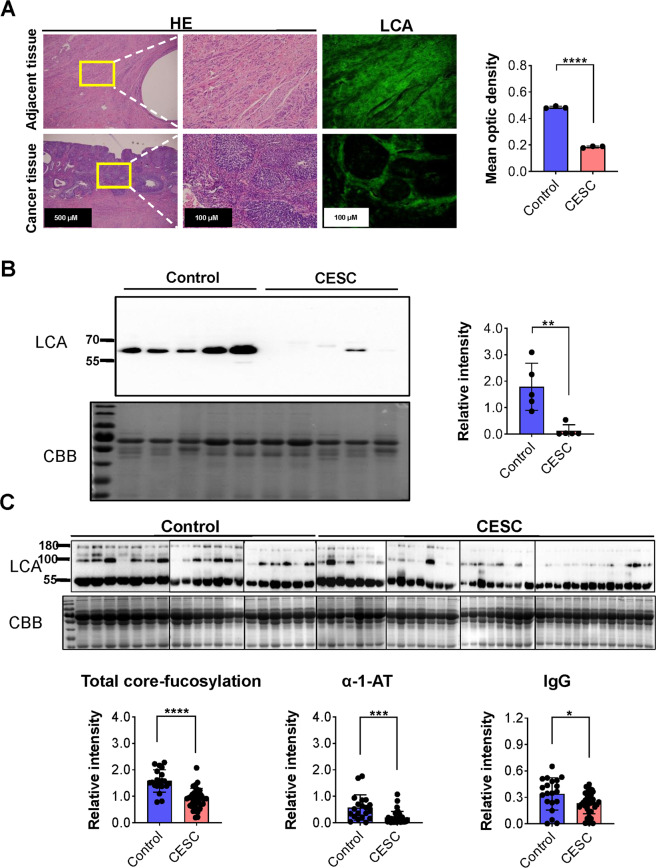
Comparison of core fucosylation between healthy volunteers and cervical cancer patients. **A** Immunofluorescence was used to analyze the core fucosylation level of cervical cancer and paracancerous tissues. **B** LCA blotting was used to compare the core fucosylation levels in exfoliated cervical cells. **C** LCA blotting was used to compare the core fucosylation levels in serum samples. *p < 0.05, **p < 0.01, ***p < 0.001, ****p < 0.0001.

Using immunofluorescence and lens culinaris agglutinin (LCA) blotting techniques, we found that the level of protein core fucosylation was significantly reduced in cancer tissue and the exfoliated cervical cells of patients with cervical cancer (Fig. 1A, B). Interestingly, we found that the core fucosylation level of serum proteins was also down-regulated when compared with the healthy controls (Fig. 1C). By means of MASS spectrum, we identified all the proteins from the SDS-PAGE, and the results were shown in Table S1. The two major core-fucosylated proteins in serum of the patients were identified as α1 trypsin inhibitor (α-1-AT, ~100 kD) and IgG (~55 kD).

### De-core fucosylation promotes the development of cervical cancer

**Fig. 2:**
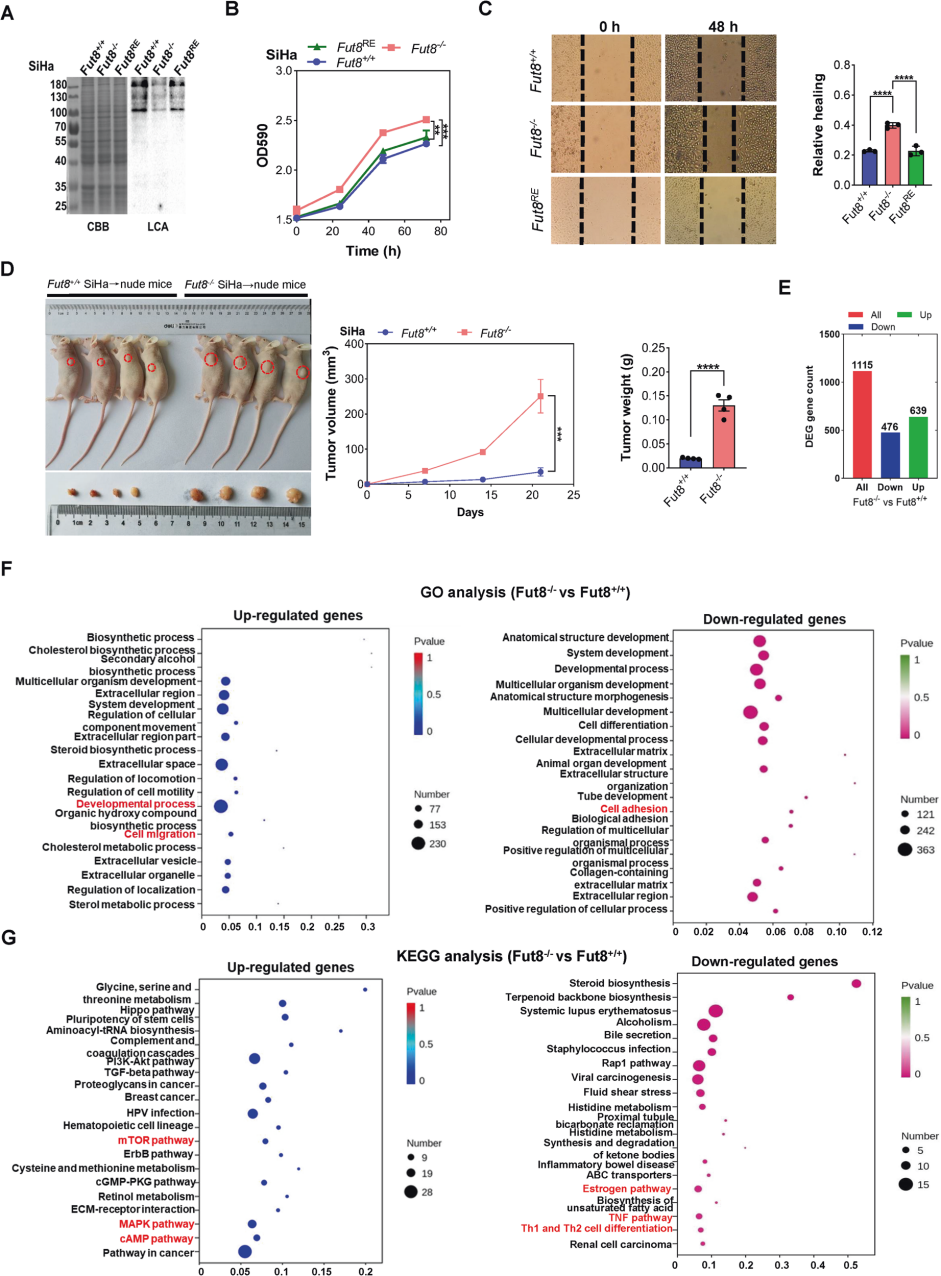
The effect of de-core fucosylation on the occurrence and development of cervical cancer. **A** LCA blotting was used to verify Fut8 gene knockout in SiHa cells. **B** An MTT assay was used to evaluate the effect of Fut8 gene knockout on the proliferation of SiHa cells. **C** A scratch assay was used to evaluate the effect of Fut8 gene knockout on the migration of SiHa cells. **D** Fut8^+/+^ and Fut8^−/−^ SiHa cells were injected into nude mice to detect tumor formation in vivo. ***p < 0.001, ****p < 0.0001. **E** Histogram of differentially expressed genes in Fut8^−/−^ SiHa cells compared to Fut8^+/+^ SiHa cells. **F** Bubble plots of GO enrichment analysis of differentially expressed genes in Fut8^−/−^ SiHa cells compared to Fut8^+/+^ SiHa cells. **G** Bubble plots of KEGG enrichment analysis of differentially expressed genes in Fut8^−/−^ SiHa cells compared to Fut8^+/+^ SiHa cells.

As core fucosylation in patients with cervical cancer was significantly reduced, we wondered whether it promotes the occurrence and development of cervical cancer. In vitro lentivirus infection was used to construct a stable Fut8-knockout and reintroduced SiHa cell line, gene knockout and reintroduced was verified by LCA blotting (Fig. 2A). Knockout of the Fut8 gene was found significantly promoted the proliferation and migration of the SiHa cells, while reintroduced of the Fut8 gene reversed the effects (Fig. 2B, C, ****p < 0.0001).

To explore the influence of de-core fucosylation on the development of cervical cancer in vivo, we further injected the Fut8^+/+^ and Fut8^−^^/−^ SiHa cells into athymic female nude mice and found that the tumor volume and weight in the Fut8^−/−^ SiHa-injected nude mice were significantly higher than that in the Fut8^+/+^ SiHa-injected nude mice (Fig. 2D, ***p < 0.001). These results suggested that the de-core fucosylation of cervical cancer cells could increase tumor growth in vivo.

Subsequently, we compared the differential expression genes of Fut8-knockout SiHa cells to that of control cells by means of transcriptome. The results showed that compared to the Fut8^+/+^ group, the Fut8^−^^/−^ group had a total of 639 genes were up-regulated, and 476 genes were down-regulated (Fig. 2E). The GO enrichment analysis showed that genes involved in the developmental process, the extracellular process, and migration capacity of Fut8^−/−^ cells were increased, while genes involved in cell adhesion and cell differentiation were reduced (Fig. 2F). In addition, the KEGG pathway analysis showed that after the Fut8 gene was knockout, the genes involved in mTOR, PI3K-Akt and MAPK signal pathway in the cervical cancer cells were significantly increased, while those involved in the TNF signal pathway and the Estrogen pathway were decreased (Fig. 2G), suggested that Fut8 may play multiple effects on the development, migration and apoptosis of cervical cancer cells.

### The vaginal microbiota of patients with cervical cancer differed significantly from healthy females

**Fig. 3:**
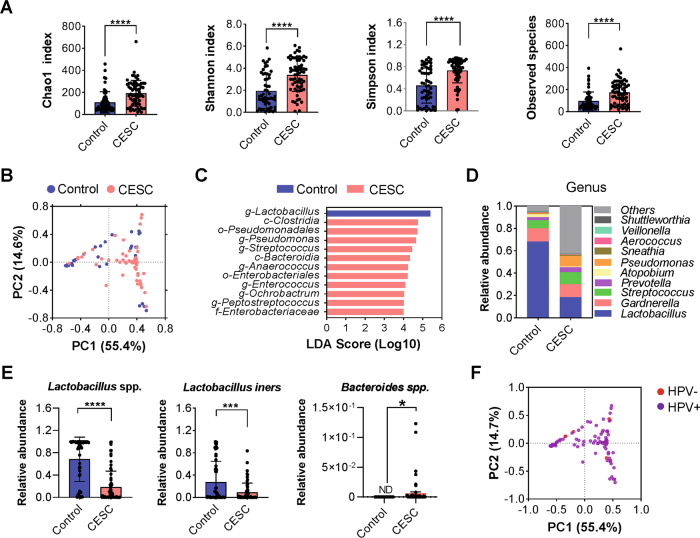
Comparison of the vaginal microbiota structures of healthy volunteers and cervical cancer patients. **A** The alpha diversity of the two groups (indicated by the Chao 1, Shannon, Simpson indices). **B** The beta diversity of the two groups (expressed based on PCA). **C** The composition of dominant microbiota in the two groups (expressed based on LEfSe analysis). **D** The relative abundance of the top ten microorganisms at the gene level in the two groups. **E** Comparison of the relative abundance of *Lactobacillus spp*. and *L. iners* in the two groups. **F** Beta diversity of the HPV- and HPV + groups (expressed based on PCA). ***p < 0.001, ****p < 0.0001.

16S rRNA sequencing was used to analyze the vaginal microbiota of healthy females and patients with cervical cancer. The alpha diversity of the vaginal microbiota in patients with cervical cancer was significantly lower than that of healthy females (Fig. 3A, ****p < 0.0001). Principal component analysis (PCA) showed significant differences in the distribution of the vaginal microbiota between groups (Fig. 3B). The results of LEfSe analysis showed that *Lactobacillus* spp. was the only dominant bacterial genus in the vaginas of healthy females, while *Clostridia* spp., *Staphylococcus* spp., and *Bacteroides* spp. were the dominant bacteria in the vaginas cervical cancer patients (Fig. 3C). At genus level, the abundance of *Lactobacillus* spp. was significantly decreased in the vagina of cervical cancer patients, while the abundance of pathogenic bacteria such as *Gardnerella* spp., and *Staphylococcus* spp. was significantly increased (Fig. 3D and S1). Further analysis of the data found a significant decrease in *L. iners* abundance in the vaginas of cervical cancer patients (Fig. 3E, ***p < 0.001). In view of the important effect of HPV infection on the occurrence and development of cervical cancer [28], we also explored the effect of HPV infection on the vaginal microbiota in healthy females and patients with cervical cancer. The results of PCA showed that HPV infection had less effect on the structure of the vaginal microbiota (Figs. 3F and S2).

### *L. iners* inhibits the proliferation and migration of cervical cancer cells

**Fig. 4:**
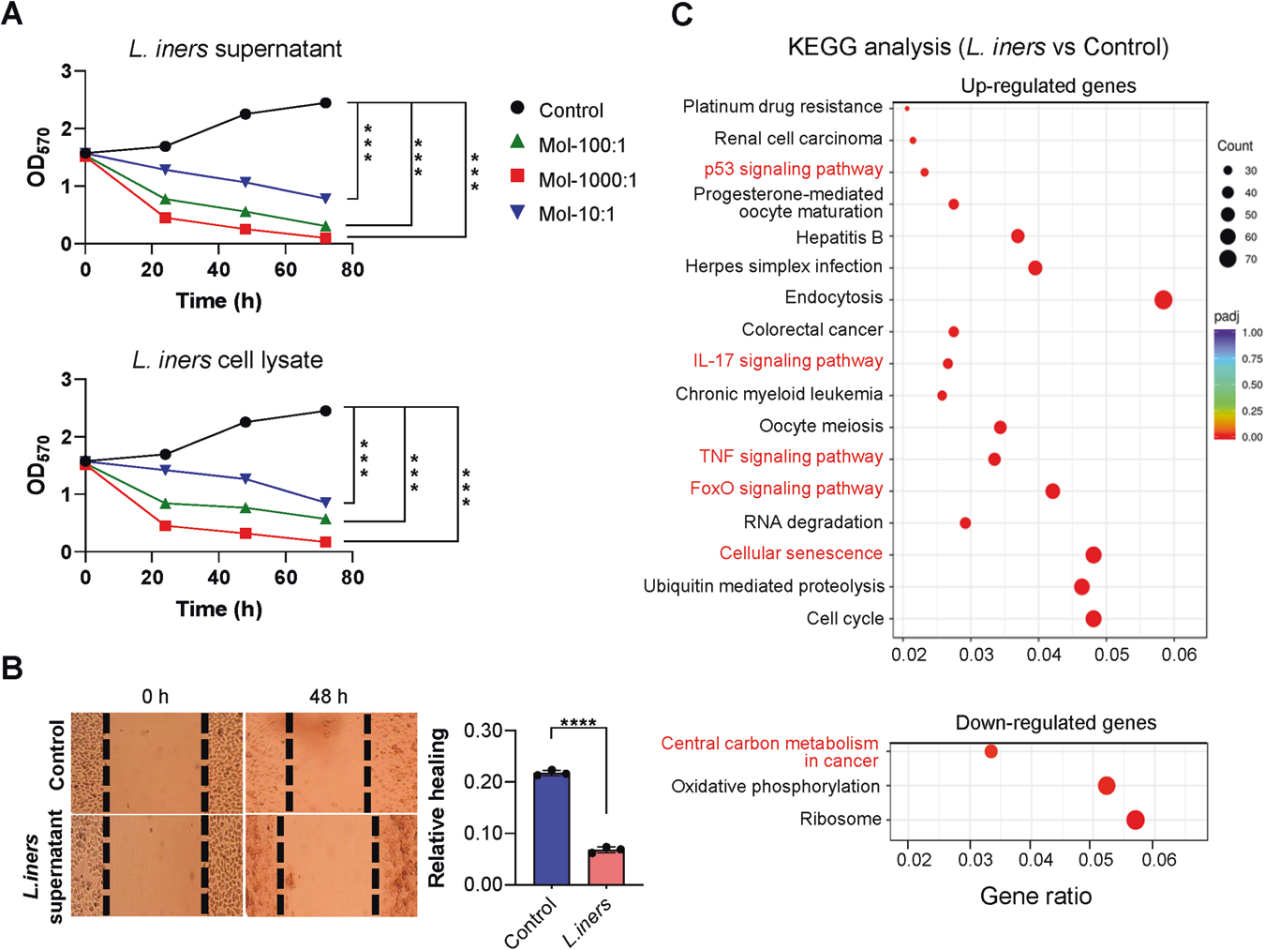
The effect of *L. iners* metabolites on the proliferation and migration of cervical cancer cells. **A** MTT assay in SiHa cells stimulated with *L. iners* metabolites and bacterial lysates. **B** Scratch assay in SiHa cells stimulated with *L. iners* metabolites. ***p < 0.001, ****p < 0.0001. **C** KEGG pathways enriched in differentially expressed genes after the treatment of cervical cancer cells with *L. iners* metabolites.

A standard *L. iners* strain (ATCC 55195) was cultured in vitro, and its metabolites and bacterial lysate were used to stimulate cervical cancer cells in vitro. Results showed that the supernatant and lysate of *L. iners* significantly inhibited the proliferation of SiHa cells, and with increasing bacterial number, the inhibitory effect increased. In addition, the scratch assay showed that the migration of the SiHa cells was significantly decreased after treatment with the *L. iners* supernatant (Fig. 4B, ****p < 0.0001). Subsequently, we stimulated SiHa cells with *L. iners* metabolites in vitro to explore the effect of *L. iners* on transcription. Interestingly, we found that *L. iners* metabolites enhanced the IL-17, p53, TNF, and FoxO signaling pathways; in addition, the *L. iners* supernatant induced cellular senescence and decrease central carbon metabolism in the cancerous cells (Fig. 4C).

### *L. iners* metabolites activate the Wnt pathway to promote the core fucosylation of epithelial cells

**Fig. 5:**
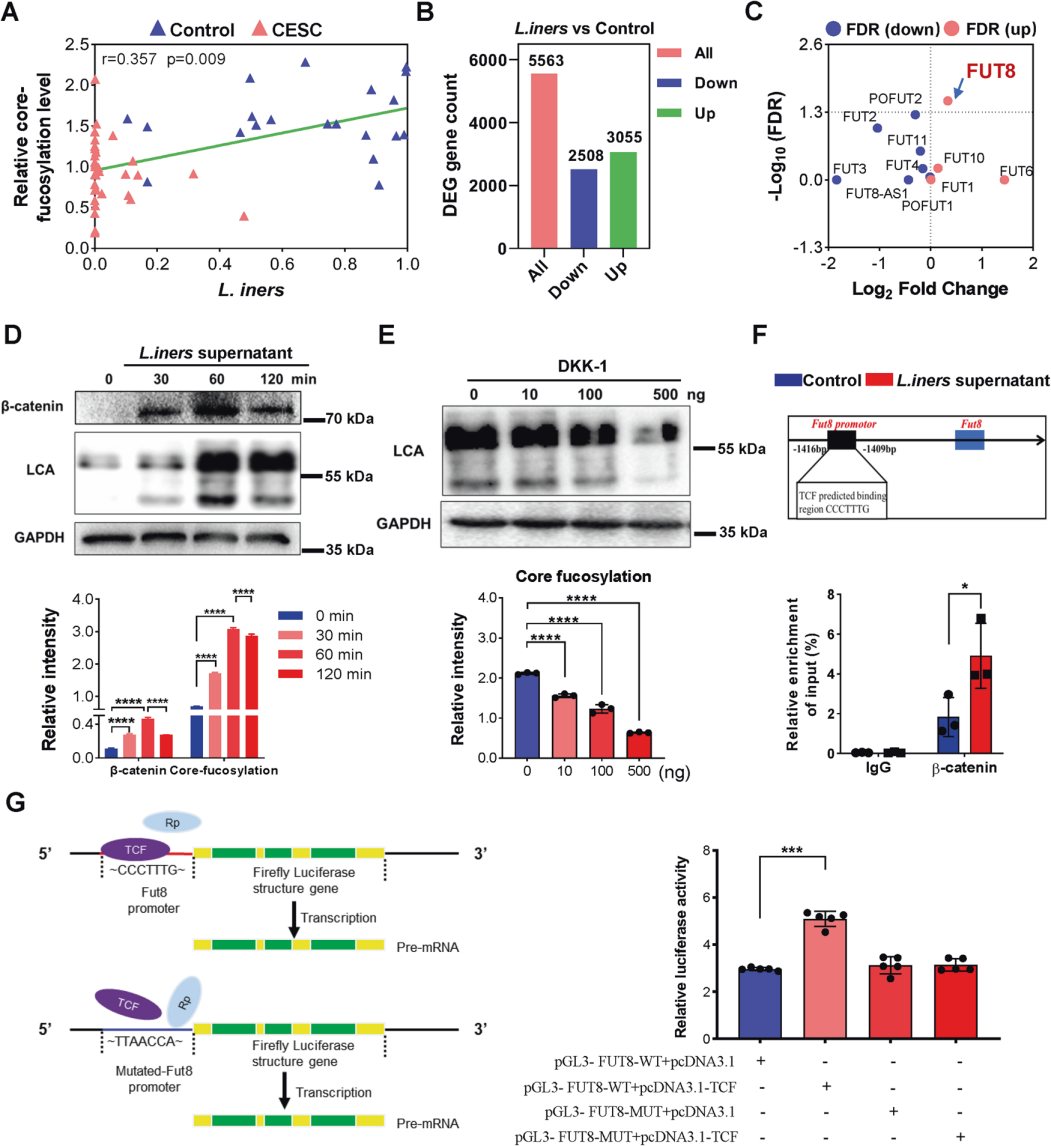
*L. iners* metabolites increased the core fucosylation of epidermal cells by activating the Wnt pathway. **A** Correlation analysis between the relative abundance of *L. iners* and core protein fucosylation. **B** Histogram of differentially expressed genes in SiHa cells stimulated with *L. iners* metabolites. **C** *L. iners* metabolites stimulated the expression of glycosylation-related genes in SiHa cells. **D** The level of core fucosylation and expression of β-catenin after treatment with *L. iners* metabolites. **E** After SiHa cells were treated with *L. iners* metabolites, DKK-1 at different doses was added, and the core fucosylation of epidermal cells was measured. **F** ChIP-qPCR was used to verify the binding of TCF/β-catenin to the promoter of the Fut8 gene. **G** Dual-luciferase reporter gene assay to verify the regulatory effect of TCF to the promoter of the Fut8 gene. *p < 0.05, ***p < 0.001, ****p < 0.0001.

Spearman correlation analysis showed that the abundance of *L. iners* in vagina of the volunteers was positively correlated with the level of serum core fucosylation (Fig. 5A, *r* **=** 0.357, ***p **=** 0.009). Subsequently, we used *L. iners* metabolites to stimulate SiHa cells in vitro to explore the effect of *L. iners* on the core fucosylation of cervical cancer cells from a transcriptomics perspective. The results showed that after *L. iners* exposure, the expression of 2508 genes decreased, while 3055 genes was up-regulated (Fig. 5B). To further clarify the influence of *L. iners* metabolites on SiHa glycosylation, all glycosyltransferase genes were separately filtered out and analyzed. Result showed that expression of only the Fut8 gene was strongly increased (Fig. 5C, *p < 0.05).

Previous research by our research group showed that the intestinal microbiota can upregulate the core fucosylation of epidermal cells by activating the Wnt pathway [29]. Thus, we speculated that *L. iners* in the vagina also activates the Wnt pathway to regulate core fucosylation in epithelial cells. Based on this notion, we found that after *L. iners* metabolite treatment, the expression of β-catenin and level of core fucosylation were significantly increased (Fig. 5D, ****p < 0.0001). KK-1 is an inhibitor of the Wnt pathway [30, 31]. In this study, we treated SiHa cells with *L. iners* metabolites and then added DKK-1 at different concentrations to the cell culture medium. As the concentration of DKK-1 increased, core fucosylation in the epidermal cells gradually decreased (Fig. 5E, ****p < 0.0001).

We further used ChIP-qPCR technology to explore the interaction between TCF/β-catenin and the Fut8 gene in the Wnt pathway. The results showed that TCF/β-catenin can bind upstream of the Fut8 promoter at bp –1416 to –1409, and *L. iners* metabolites can significantly enhance the transcriptional activity of the Fut8 gene (Fig. 5F, *p < 0.05). On the basis of CHIP-qPCR results, we constructed luciferase vectors harboring the wild type and mutated Fut8 promoter region (the binding motif for TCF was mutated). The Dual-Luciferase Reporter Assay results indicate that the transcription factor TCF can bind to the promoter region of FUT8 gene and initiate its transcription, which is expressed as a significant increase in luciferase activity (Fig. 5G, ***p < 0.001), while the TCF failed to activate the Fut8 transcription when the predicted binding site in Fut8 promoter was mutated from CCCTTTG into TTAACCA, which resulted in no significant change in the luciferase activity.

### *L. iners* metabolizes activate the Wnt pathway through the lactate-Gpr81 complex

**Fig. 6:**
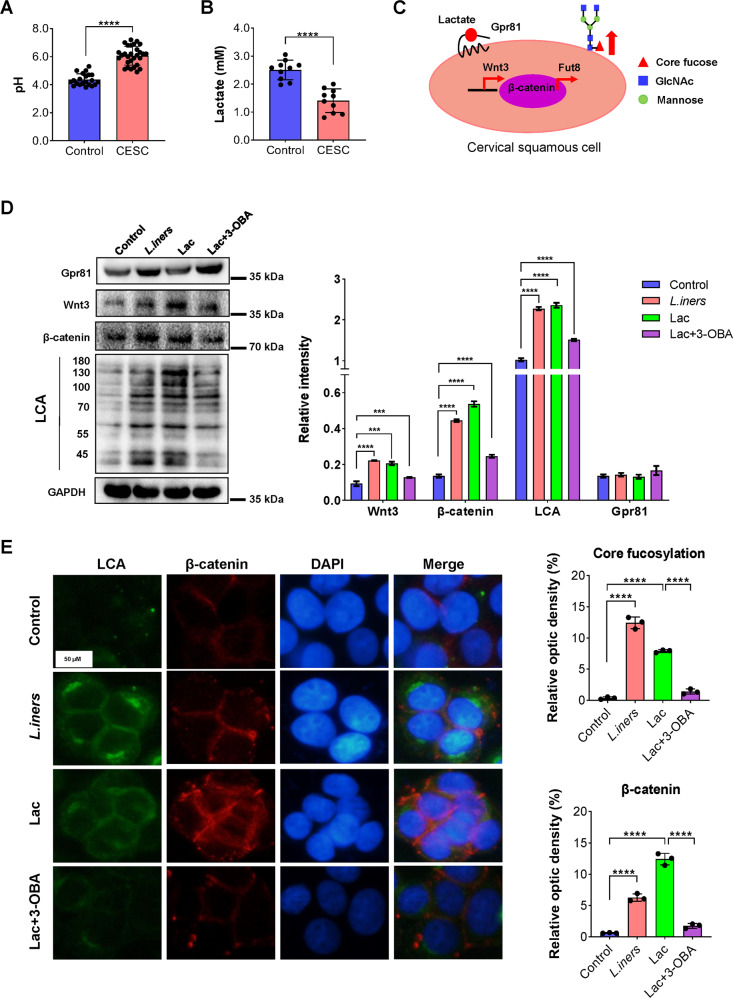
The *L. iners* metabolite lactate activates the Wnt pathway through the lactate-Gpr81 complex to increase the core fucosylation of epidermal cells. **A** Comparison of vaginal pH in healthy volunteers and cervical cancer patients. **B** Comparison of vaginal lactate levels in the two groups. **C** Schematic diagram of the effect of lactate. **D** After SiHa cells were treated with *L. iners* metabolites, lactate and lactate and 3-OBA, the level of core fucosylation and expression of β-catenin, wnt3, and Gpr81 were determined. **E** Cellular immunofluorescence experiments were used to analyze the level of core fucosylation and expression of β-catenin after SiHa cells were treated with *L. iners* metabolites, lactate and lactate and 3-OBA. ***p < 0.001, ****p < 0.0001.

*Lactobacillus spp*. can produce lactic acid to maintain the acidic vaginal environment [32, 33]. We found that the vaginal pH of the cervical cancer patients was significantly higher than that of the healthy females (Fig. 6A, ****p < 0.0001), while the lactate content was significantly reduced in the cervical cancer patients (Fig. 6B, ****p < 0.0001). We speculated that the presence of *L. iners* metabolites, such as lactate, in the vagina can activate the Wnt pathway through the lactate-Gpr81 complex to regulate the core fucosylation of epidermal cells (Fig. 6C) [34]. To verify this hypothesis, we stimulated SiHa cells with *L. iners* metabolites, lactate, and lactate with 3-OBA and detected levels of the lactate receptors Gpr81, wnt3, and β-catenin and core fucosylation in epidermal cells. The results showed that the *L. iners* supernatant and lactate increased the levels of wnt3, β-catenin, and core fucosylation in SiHa cells, but after the addition of 3-OBA, the wnt3, β-catenin, and core fucosylation levels in the SiHa cells were significantly decreased, indicating that lactate is a key *L. iners* metabolite that can activate the Wnt pathway (Fig. 6D, ***p < 0.001, ****p < 0.0001). Cellular immunofluorescence experiments also confirmed that the *L. iners* metabolite lactate could increase the expression of β-catenin and upregulate the core fucosylation of epidermal cells. However, once 3-OBA was added, the levels of β-catenin and core fucosylation were significantly decreased (Fig. 6E, ****p < 0.0001).

## Discussion

In-depth exploration of the pathogenesis of cervical cancer would have a positive and important impact on the diagnosis and treatment of this disease [35, 36]. As a basic physical barrier, vaginal mucosal epithelial cells are critical for maintaining the homeostasis of the female vaginal microenvironment [37]. Core fucosylation is one of the most important glycosylation modifications of vaginal mucosal epithelial cells [38]. Studies have found that the fucosylation of intestinal mucosal epithelial cells is closely related to microorganism colonization [39]. Terahara et al. [40] also found that changes in intestinal commensal microbiota can cause corresponding changes in local fucosylation levels. According to reports, patients with cervical cancer exhibit abnormal fucosylation levels [24, 25] and disorder of the vaginal microbiota [41], but the correlation between abnormal fucosylation and disorder of the vaginal microbiota and their roles in the occurrence and development of cervical cancer have not been reported. For the first time, we systematically analyzed the correlation and relationship between disorder of the vaginal microbiota and abnormal core fucosylation in patients with cervical cancer, providing new insight into the prevention, diagnosis and treatment of cervical cancer.

The core fucosyltransferase (Fut8), which catalyzes the transfer of GDP-fucose to GlcNAc adjacent to Asn in the N-glycan of a protein, is closely related to the occurrence and development of a variety of tumors [22, 23, 42–44]. In this study we found that cervical cancer patient serum, exfoliated cervical cells, and cancer tissues all showed significantly reduced core fucosylation level, and Jin et al [24, 25]. also presented similar results. To further determine the biological significance of the protein core fucosylation, we constructed a Fut8 gene-knockout cell line and Fut8 gene-knockout mouse model. The proliferation and migration of cervical cancer cells was significantly enhanced after the Fut8 gene was knocked out, while reintroduced of the Fut8 gene reversed the effects. In addition, the tumor weight and volume were significantly greater in the 0Fut8^−/−^ SiHa cell-injected nude mice when compared with those injected with Fut8^+/+^ SiHa cells, revealing that the core fucosylation has a protective effect against cervical cancer development. Additionally, the transcriptome analysis showed that knockout of Fut8 gene could enhance cell migration and decrease cell adhesion. It could enhance the mTOR, cAMP and MAPK signaling pathways. Studies have reported that activation of the mTOR, cAMP and MAPK signaling pathways plays an important role in the proliferation and migration of cancer [45–47]. On the contrary, it could reduce the TNF signaling pathways, which had been shown to promote tumor apoptosis when activated [48]. The above results suggested that when Fut8 gene was knockout, the proliferation and migration capacity of cervical cancer cells have increased significantly, and the apoptosis ability was weakened, it will accelerate the occurrence and development of cervical cancer.

Disorder of the vaginal microbiota is closely related to the occurrence and development of cervical cancer. We found that *Lactobacillus spp*. were the only dominant bacteria in the vaginas of healthy females, while the vaginal microbiota of cervical cancer patients showed a significantly increased species; a significantly increase in abundance of *Gardnerella spp*., *Staphylococcus spp*., *Bacteroides spp*. and other pathogenic bacteria was detected. This may be because the vaginal pH of patients with cervical cancer is significantly higher than that of healthy females, which is conducive to the growth of pathogenic bacteria under a variety of conditions [49, 50]. Studies [51, 52] have found that dysbiosis of vaginal microbiota can induce viral infection, immune escape, DNA damage, and an inflammatory response in the local microenvironment. The combined action of these factors can induce cell canceration. *L. crispatus*, *L. gasseri*, *L. iners*, and *L. jensenii* were reported to be the four most common types of *Lactobacillus* spp. in the vaginas of healthy females [53]. We found that the main *Lactobacillus* species in the vaginas of healthy Chinese females was *L. iners*, and its metabolites could significantly inhibit the growth of cervical cancer cells. Wang et al. [54] and Li et al. [55] have reported similar results. The transcriptome analysis showed that *L. iners* metabolites could enhance the IL-17, p53, TNF, and FoxO signaling pathways. Activation of these signaling pathways plays an important role in the prevention and treatment of cancer [48, 56, 57]. In addition, we found that *L. iners* metabolites could inhibit the growth of *B. fragilis*, which can promote the proliferation and migration of cervical cancer cells, under acidic conditions (Fig. S3). Therefore, *Lactobacillus spp*. have important application prospects for inhibiting the occurrence and development of cervical cancer.

We further stimulated the cervical cancer cells with *L. iners* metabolites in vitro and found that the *L. iners* metabolites activated the Wnt pathway to promote the core fucosylation of epithelial cells. Once Wnt pathway inhibitors were added, core fucosylation in the epithelial cells decreased. To further verify that the metabolites of *L. iners* indeed promote core fucosylation through the Wnt pathway, we used ChIP-qPCR technology [58, 59]. The results showed that when the Wnt pathway was activated, the key molecule β-catenin entered the nucleus from the cytoplasm and bound TCF to form the TCF/β-catenin complex, which then bound upstream of the Fut8 gene promoter at bp −1416 to −1409. This binding further promoted transcription of the Fut8 gene and increased the core fucosylation of epithelial cells. To further verify the regulatory effect of TCF on the Fut8, we used the Dual-Luciferase Reporter Gene Assay, which is a report system for detecting Firefly and Renilla luciferase activity based on fluorescein as substrates [60]. The results indicated that the transcription factor TCF can activate the Fut8 gene transcription, which is expressed as a significant increase in luciferase activity, while the TCF cannot activate the Fut8 transcription when the predicted binding site was mutated, resulted in no significant change in the luciferase activity.

However, the identity of the *L. iners* metabolites and how they activate the Wnt pathway remain to be studied in depth. According to reports, the vaginal pH is ≤4.5 in healthy females, while that in patients with cervical cancer is >4.5 [61, 62]. The acidic vaginal environment is mainly maintained by the catabolism of carbohydrates by *Lactobacillus*, producing lactic acid [63]. Therefore, we speculate that lactic acid activates the Wnt pathway. In addition, studies have shown that formation of a complex between lactate and Gpr81, a receptor for lactate, can activate the Wnt pathway [64]. Based on this finding, we stimulated cervical cancer cells with *L. iners* metabolites lactate and lactate and 3-OBA and found that *L. iners* metabolites and lactate promoted expression of the Wnt pathway-related proteins wnt3 and β-catenin and the level of core fucosylation in epithelial cells. When the lactate antagonist 3-OBA was added, the expression of these proteins was significantly reduced, indicating that lactate among the *L. iners* metabolites can activate the Wnt pathway through the lactate-Gpr81 complex, thereby promoting the core fucosylation of epidermal cells. However, interestingly, the expression of Gpr81 was not increase after stimulation with lactate, which may be because its confirmation changes after it binds lactate [65].

**Fig. 7:**
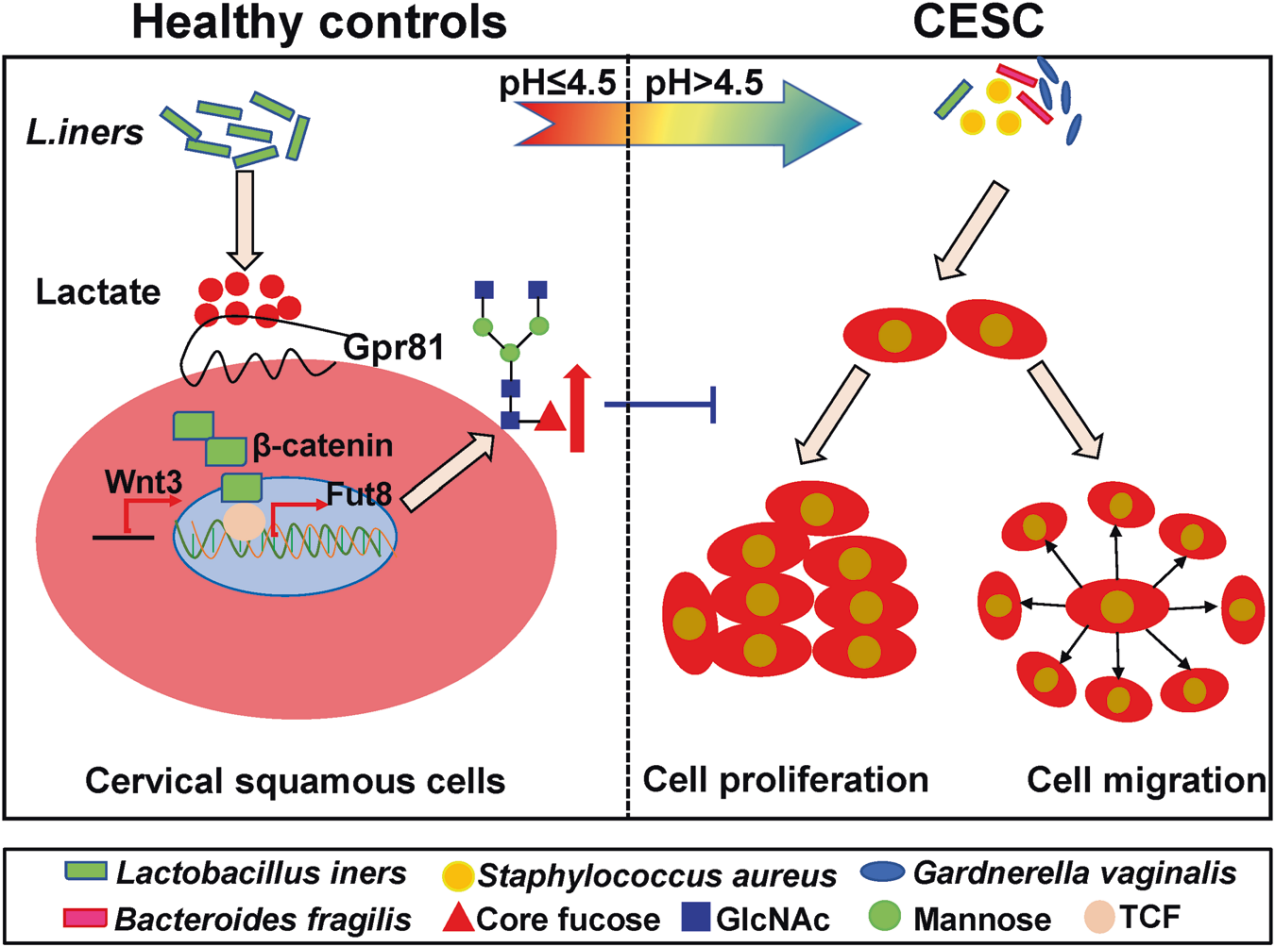
The molecular mechanism by which *Lactobacillus spp.* regulate the core fucosylation of epithelial cellsthrough the Wnt pathway. *Lactobacillus iners* can secrete lactate to increase the level of core fucosylation of epithelial cells by activating the Wnt pathway, which inhibits the proliferation and migration of cervical cancer cells.

In summary, results of this study proved that the vaginal dysbiosis is closely related to core fucosylation levels of the cervical cancer patients. *L. iners* metabolite lactate can activate the Wnt pathway through the lactate-Gpr81 complex, which increases the level of core fucosylation in epidermal cells, results in inhibiting of the proliferation and migration of cervical cancer cells (Fig. 7). This study have important biological significance for the prevention and treatment of cervical cancer.

## Supplementary information

Checklist

Supplementary information

Figure S1

Figure S2

Figure S3

Table S1

## Data Availability

All data needed to evaluate the conclusions in the paper are present in the paper and/or the Supplementary Materials. Additional data related to this paper may be requested from the authors.
