# Supplementary material for: Lactobacillus spp. create a protective micro-ecological environment through regulating the core fucosylation of vaginal epithelial cells against cervical cancer
Source: Cell Death Dis. 2021 Nov 20;12(12):1094. doi: 10.1038/s41419-021-04388-y (PMC8604912; doi:10.1038/s41419-021-04388-y)
Supplement: Supplementary file 2 — Supplementary information [file 41419_2021_4388_MOESM2_ESM.docx]

**Supplementary information**

**Figure S1** The relative abundance of the top twenty microorganisms at the phylum and family levels in healthy volunteers and cervical cancer patients.

**Figure S2** Comparison of the vaginal microbiota structure in the HPV-negative and HPV-positive groups. **A** The alpha diversity of the two groups (indicated by the Chao 1, Shannon, and Simpson indices). **B** The beta diversity of the two groups (expressed based on NMDS). **C** The relative abundance of the top twenty microorganisms at the gene and family levels in the two groups.

**Figure S3** *B. fragilis* promotes the proliferation and migration of cervical cancer cells. **A** The relative abundance of *Bacteroides spp.* and *B. fragilis* in healthy volunteers and cervical cancer patients. **B** The growth of *B. fragilis* under different pH conditions. **C** MTT assay in SiHa cells stimulated with *B. fragilis* supernatant. D Scratch assay in SiHa cells stimulated with *B. fragilis* supernatant.

**Table S1** After the serum samples were subjected to SDS-PAGE electrophoresis, gel strips were used to analyse proteins of various molecular weights by mass spectrometry.
