# Supplementary figures and images for: Lactobacillus spp. create a protective micro-ecological environment through regulating the core fucosylation of vaginal epithelial cells against cervical cancer
Source: Cell Death Dis. 2021 Nov 20;12(12):1094. doi: 10.1038/s41419-021-04388-y (PMC8604912; doi:10.1038/s41419-021-04388-y)

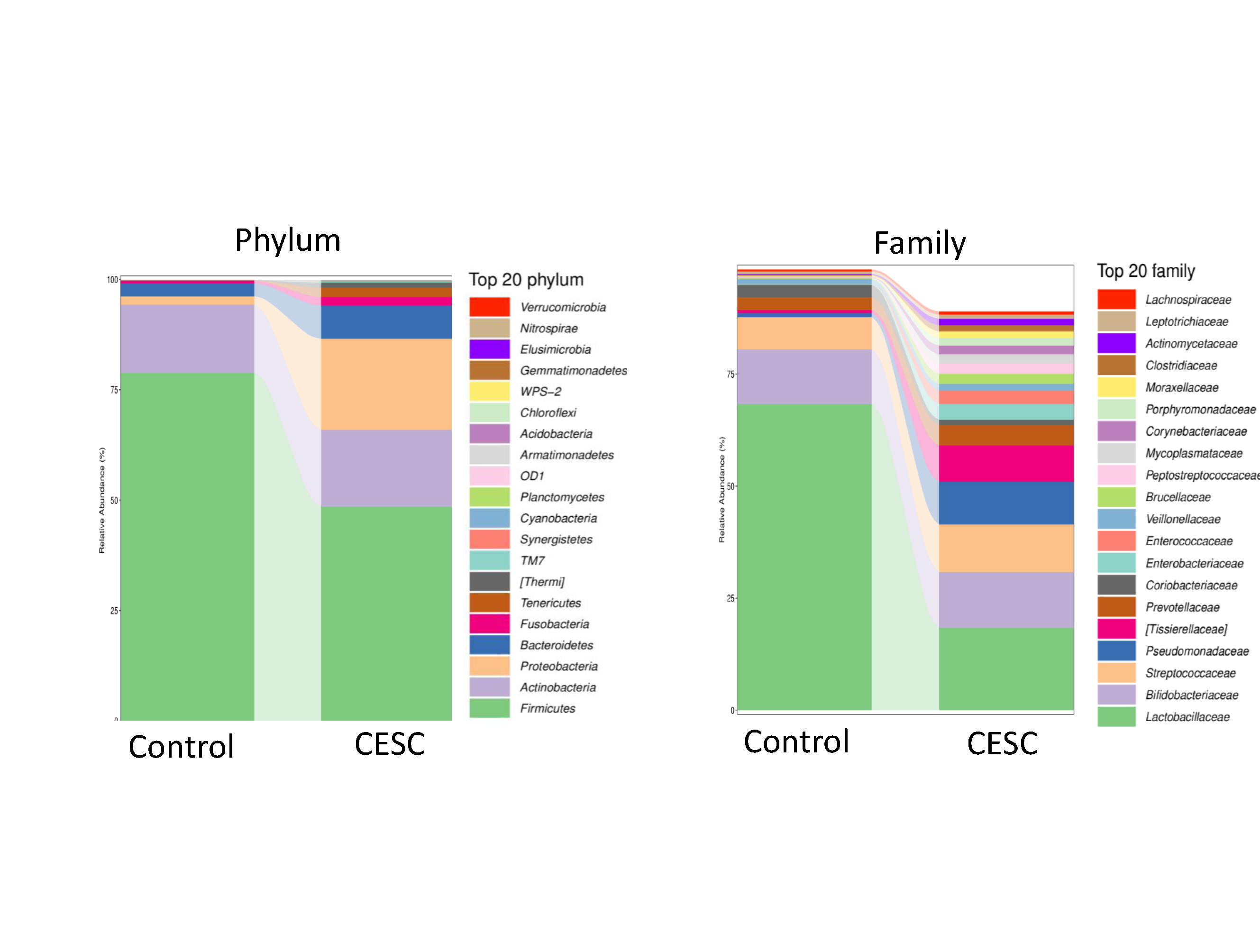

Supplement: Supplementary file 3 — Figure S1 [file 41419_2021_4388_MOESM3_ESM.tif]

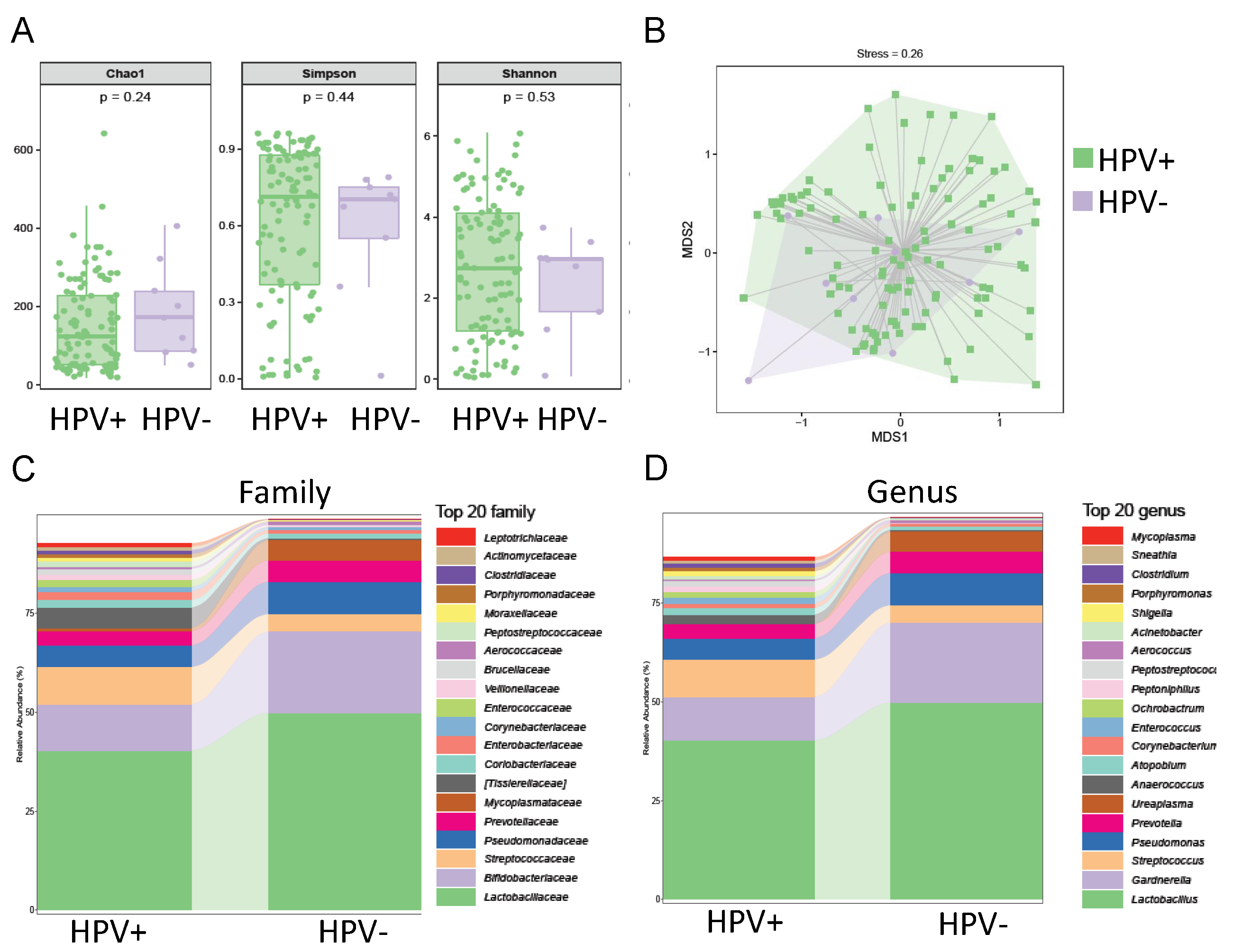

Supplement: Supplementary file 4 — Figure S2 [file 41419_2021_4388_MOESM4_ESM.tif]

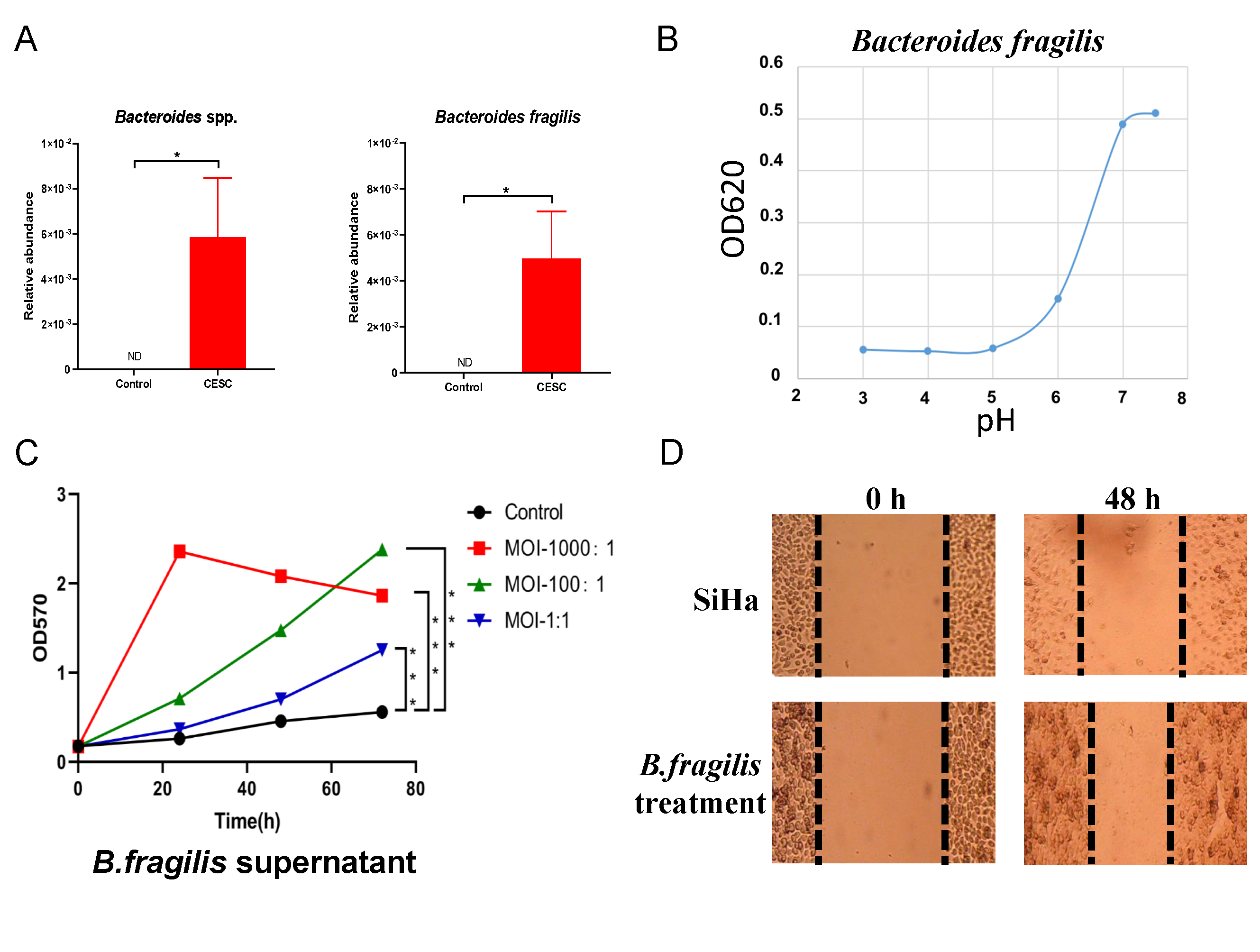

Supplement: Supplementary file 5 — Figure S3 [file 41419_2021_4388_MOESM5_ESM.tif]
